# Monitoring of semiconductor manufacturing process on Bayesian AEWMA control chart under paired ranked set sampling schemes

**DOI:** 10.1038/s41598-023-49843-2

**Published:** 2023-12-19

**Authors:** Yuzhen Wang, Imad Khan, Muhammad Noor-ul-Amin, Salman A. AlQahtani, Bakhtiyar Ahmad

**Affiliations:** 1 School of Science, Xi’an Technological University, Xi’an, 710032 China; 2 Department of Statistics, Abdul Wali Khan University Mardan, Mardan, Pakistan; 3 Department of Statistics, COMSATS University Lahore, Lahore, Pakistan; 4 Computer Engineering Department, College of Computer and Information Sciences, King Saud University, Riyadh, Saudi Arabia; 5 Higher Education Department Afghanistan, Kabul, Afghanistan

**Keywords:** Mathematics and computing, Theory and computation

## Abstract

Quality control often employs memory-type control charts, including the exponentially weighted moving average (EWMA) and Shewhart control charts, to identify shifts in the location parameter of a process. This article pioneers a new Bayesian Adaptive EWMA (AEWMA) control chart, built on diverse loss functions (LFs) such as the square error loss function (SELF) and the Linex loss function (LLF). The proposed chart aims to enhance the process of identifying small to moderate as well as significant shifts in the mean, signifying a notable advancement in the field of quality control. These are implemented utilizing an informative prior for both posterior and posterior predictive distributions, employing various paired ranked set sampling (PRSS) schemes. The effectiveness of the suggested chart is appraised using average run length (ARL) and the standard deviation of run length (SDRL). Monte Carlo simulations are employed to contrast the recommended approach against other control charts. The outcomes demonstrate the dignitary performance of the recommended chart in identifying out-of-control signals, especially applying PRSS designs, in comparison to simple random sampling (SRS). Finally, a practical application was conducted in the semiconductor manufacturing context to appraise the efficacy of the offered chart using various paired ranked set sampling strategies. The results reveal that the suggested control chart performed well in capturing the out-of-control signals far better than the already in use control charts. Overall, this study interposes a new technique with diverse LFs and PRSS designs, improving the precision and effectiveness in detecting process mean shifts, thereby contributing to advancements in quality control and process monitoring.

## Introduction

Statistical Process Control (SPC) is a critical quality management tool employed in various industries to supervise, regulate, and improve production processes. By using statistical methodologies, SPC ensures the continuous monitoring of manufacturing operations, ensuring their effective and reliable function within predefined quality benchmarks. It involves data collection, analysis, and interpretation to identify variations and trends within the production process. Utilizing control charts (CCs), and other statistical tools, SPC aids in the timely identification of potential deviations from normal patterns, facilitating swift corrective measures to maintain desired quality levels. SPC significantly contributes to defect reduction, enhanced production efficiency, and the overall improvement of product quality, resulting in increased customer satisfaction and reduced operational costs. A CC is a fundamental component of SPC that facilitates the ongoing monitoring and evaluation of the stability and performance of manufacturing or business processes. It visually represents process data, enabling the identification of variations and trends that could potentially impact output quality. By plotting data points on a graph with predetermined control limits, it assists in recognizing common sources of variation, such as random fluctuations, as well as special causes like defects or errors. CCs enable organizations to distinguish between normal process variations and those requiring corrective actions, thereby ensuring consistent product quality and preventing defects. The effective utilization of control charts enables businesses to make informed, data-driven decisions, improve process efficiency, and achieve higher levels of customer satisfaction. Renowned engineer and statistician Walter Shewhart^1^ is widely recognized for pioneering the concept of memoryless type CCs. These innovative CCs are specifically engineered to swiftly and accurately identify noteworthy shifts within the production process. They achieve this by solely utilizing the most recent sample data, allowing for precise real-time monitoring and prompt corrective actions in industrial settings. Conventional memory type CCs, exemplified by the cumulative sum (CUSUM) and EWMA CCs, as proposed by Ref.^2,3^, are primarily applying for efficiently managing and monitoring small-to-moderate variations within processes. These traditional memory type CCs have undergone ongoing refinements and advancements, as evidenced by the developments presented in research works such as Refs.^4–8^. Haq et al.^9^ focuses on adaptive memory-type CCs, including AEWMA and dual CUSUM, demonstrating their superior performance in detecting mean shifts. The proposed AEWMA chart utilizes an unbiased mean shift estimator and dynamically adjusts the smoothing constant, outperforming existing AEWMA, adaptive CUSUM, and Shewhart-CUSUM charts. An illustrative example clarifies the functionality of the CCs. Sparks^10^ studied and proposed efficient CUSUM procedures for detecting a range of unknown location shifts, utilizing multiple CUSUM statistics with varied resetting boundaries, and an adaptive CUSUM statistic. Comparative analysis using the ARL demonstrates the relative performance of the procedures, supported by various applications. Capizzi and Masarotto^11^ propose an adaptive EWMA CC that balances the detection of small and large shifts, addressing limitations of a single EWMA chart. It combines Shewhart and EWMA features, offering improved protection against shifts of varying sizes, as demonstrated through average run length profiles. The current literature extensively covers various research efforts aimed at examining the utilization and effectiveness of adaptive cumulative sum (ACUSUM) and AEWMA CCs in identifying the variations in the process parameter. Key studies, such as those cited as Refs.^12–16^, contribute significantly to the comprehensive comprehension and assessment of these CC methodologies. Zaman et al.^17^ emphasizes the need to monitor both small and large shifts in production processes. It introduces an adaptive EWMA method with Huber and Tukey's bi-square functions, effectively monitoring various shifts, supported by real-world data analysis and performance metrics. The conventional studies have largely relied on standard methodologies that focus on analyzing sample data in isolation, disregarding any existing prior knowledge. On the other hand, the Bayesian methodology uniquely integrates both the available sample data and pre-existing information, consistently updating and refining the analysis to generate a posterior (P) distribution. This dynamic and iterative process enables a more comprehensive and nuanced estimation procedure, ultimately reinforcing the resilience and reliability of the analysis and its outcomes. Girshick and Rubin^18^ are the first who researched the notion of Bayesian CC for location parameter. Saghir et al.^19^ introduced a Bayesian CC that utilizes the P distribution to identify fluctuations in location parameter. Their method considers different LFs, allowing flexibility in capturing the underlying process characteristics. On a similar note, Riaz et al.^20^ extended the Bayesian framework by proposing a Bayesian EWMA control chart. This CC incorporates the P and posterior predictive (PP) distributions and accommodates numerous LFs. Moreover, they explored the performance of the chart utilizing both informative and non-informative priors. Riaz et al.^21^ highlights the dominance of frequentist approaches in process monitoring, although Bayesian methodology proves advantageous, particularly with limited phase-I datasets. The study emphasizes the necessity for a corrected design of Bayesian CCs to achieve the desired in-control performance, especially with various LFs. Furthermore, the predictive CC is also introduced with simulations and real data examples illustrating the concepts. Noor et al.^22^ introduces Bayesian CCs for non-normal life time distributions, employing various LFs and transforming Exponential distributions. Run length profile are used for performance evaluation which indicates that the Weibull distribution demonstrates the most valuable results, validated by extensive simulations and a real-world case study. Noor-ul-Amin and Noor^23^ introduces a novel AEWMA CC in Bayesian theory, integrating Shewhart and EWMA CCs for effective monitoring of process mean under different LFs. Performance evaluation involves ARL and SDRL, with comparisons made against existing Bayesian EWMA CCs. Asalam et al.^24^ presents  a novel Bayesian Modified-EWMA chart employing four  LFs and a conjugate prior distribution demonstrating better efficiency in identifying slight to moderate deviations when compared to current charts. Demonstrated through practical cases: monitoring the mechanical industry's reaming process and sports industry's golf ball performance. Lin et al.^25^ developed the applicability of manufacturing industry quality control methods to service quality measurement in the automated service sector. It addresses challenges unique to service processes through a Bayesian Phase II EWMA CC, demonstrating robust performance via simulated and practical examples. Khan et al.^26^ studied a new Bayesian HEWMA CC is proposed using RSS strategies, with an informative prior and various LFs. Extensive Monte Carlo simulations demonstrate its superior performance, as evidenced by ARL and SDRL. Liu et al.^27^ introduces a novel Bayesian CC that utilizes different LFs and PRSS schemes, showcasing superiority in detecting out-of-control indications, especially applying PRSS compared to SRS. Monte Carlo simulations validate its effectiveness and a real-life semiconductor manufacturing application confirms its superiority over existing control charts, offering an improved approach for identifying process mean shifts. The utilization in the semiconductor manufacturing hard-bake process demonstrates the increased sensitivity of the offered HEWMA chart with RSS designs, in contrast to other CCs utilizing SRS.

The objective of this article is to introduce a new Bayesian chart that integrates distinct paired RSS (PRSS) methodologies, including PRSS, quartiles PRSS (QPRSS), and extreme PRSS (EPRSS). The methodology includes the integration of pertinent prior distributions and the P , which are established using the chosen LFs. The effectiveness evaluation of the offered CC is conducted using run length results. The study is organized into several sections, with Section "Bayesian approach" introducing Bayesian methodology, Section "Paired ranked set sampling" examining different PRSS schemes, Section "Simulation study" detailing the design of the Bayesian AEWMA CC, Section "Results and discussions" presenting results and discussions, Section "Real data applications" discussing real-world applications, and Sect. “Conclusion” offering concluding remarks.

## Bayesian approach

A foundational framework for statistical inference, the Bayesian approach places a strong emphasis on the representation and manipulation of uncertainty through probabilities. The Bayesian method, named after Reverend Thomas Bayes, employs Bayes' theorem to calculate event probabilities based on prior knowledge. It combines prior beliefs with observed evidence to generate posterior probabilities, enabling dynamic belief updating. It underpins statistical inference and decision-making, enhancing understanding of complex systems. To draw conclusions about unknown quantities, the Bayesian paradigm incorporates both observed data and prior beliefs. The Bayesian approach approaches parameters as random variables with their own probability distributions, in contrast to frequentist statistics, which treats parameters as fixed but unknown values. This enables the measurement of the estimating process's uncertainty. Because they offer a flexible and natural way to incorporate past knowledge into statistical modeling and analysis, Bayesian methods are widely used in many fields, including machine learning, data analysis, and decision making under uncertainty. In situations where data is scarce or noisy, they offer a potent tool for well-informed decisions and forecasts. Additionally, a dynamic and iterative learning process is made possible by the Bayesian approach, which permits beliefs to be updated in response to new data. In the context of statistical analysis, the variable under consideration *X* represents an under control process with parameters θ and $\delta^{2}$. A normal prior distribution is chosen with parameters $\theta_{0}$ and $\delta_{0}^{2}$ to express initial beliefs or knowledge about these parameters prior to any data observation is given by:

1 $$p\left( \theta \right) = \frac{1}{{\sqrt {2\pi \delta_{0}^{2} } }}\exp \left\{ { - \frac{1}{{2\delta_{0}^{2} }}\left( {\theta - \theta_{0} } \right)^{2} } \right\}.$$

When there is little or no previous knowledge about an unknown population parameter, Bayesian analysis frequently applies a non-informative prior, which is typically has a negligible impact on the prior distribution. In response to this, Jeffrey^28^ formulated a prior distribution which is directly proportional to the Fisher information matrix, thereby addressing this particular scenario. The probability function is defined as $p\left( \theta \right) \propto \sqrt {I\left( \theta \right)}$ where, $I\left( \theta \right)$ is known as Fisher information matrix. This enables the analysis to incorporate any accessible information on the parameter.

The Bayesian P distribution, updates our knowledge of parameters of interest by fusing prior beliefs with the likelihood function derived from the analyzed data. Considering both past knowledge and recent evidence, it represents the refined beliefs about these parameters. In order to enable a methodical approach to statistical decision-making by combining both prior beliefs and observed data, the P distribution is a crucial part of Bayesian inference. The $p\left( {\theta |x} \right)$ is given as $p\left( {\theta |x} \right) = \frac{{p\left( {x|\theta } \right)p\left( \theta \right)}}{{\int {p\left( {x|\theta } \right)p\left( \theta \right)d\theta } }}$. The predictive distribution in Bayesian statistics, is a key tool for predicting upcoming observations by fusing prior assumptions about parameters with likelihood derived from data. The Bayes theorem is used to update our knowledge of unknown quantities in light of fresh evidence. With parameter uncertainty and data variability taken into account, it computes the probability distribution of future observations y given observed data x. This method is useful in areas like machine learning, econometrics, and uncertainty-aware decision-making because it enables the quantification of uncertainty in complex data scenarios.The predictive distribution ensures a principled approach to prediction, integrating prior knowledge with observed data for well-informed decision-making. the $p\left( {y|x} \right)$ is mathematically described as

2 $$p\left( {y|x} \right) = \int {p\left( {y|\theta } \right)p\left( {\theta |x} \right)d\theta } .$$

### Squared error loss function

In the context of the Bayesian approach, The SELF is a metric that assesses the discrepancy between the estimated and true parameters. It serves as a way to evaluate the accuracy of an estimator by considering the squared difference between the true value and the estimated value. The SELF is a fundamental component in Bayesian decision theory, where it helps to assess the quality of estimators and aids in making decisions. Specifically, it helps in quantifying the loss incurred due to the incongruity between the estimated and true values, with the aim of minimizing this loss in the decision-making process. Gauss^29^ suggested a SELF and mathematically described as

3 $$L\left( {\theta ,\,\hat{\theta }} \right) = \left( {\theta - \hat{\theta }} \right)^{2} .$$

Using SELF the Bayes estimator is mathematized as:

4 $$\hat{\theta } = E_{\theta /x} \left( \theta \right).$$

### Linex loss function

Within the Bayesian framework, the LLF, asymmetric measurement, evaluates the distinction between the actual and the estimated parameter. It integrates exponential and linear components, enabling the evaluation of accuracy with non-uniform preferences. This characteristic foster adaptability in decision-making and estimation, aligning with specific preferences and priorities in the Bayesian approach. Varian^30^ introduced an asymmetric LLF. The estimation method for the location parameter under the LLF can be described as follows:

5 $$L\left( {\theta ,\hat{\theta }} \right) = \left( {e^{{c\left( {\theta - \hat{\theta }} \right)}} - c\left( {\theta - \hat{\theta }} \right) - 1} \right).$$

Utilizing LLF, the Bayes estimator is given mathematically as

6 $$\hat{\theta } = - \frac{1}{c}InE_{\theta /x} \left( {e^{ - c\theta } } \right).$$

## Paired ranked set sampling

Muttlak^31^ is recognized as the pioneer of the paired RSS (PRSS) method. This technique involves selecting a subset of population units for ranking, and instead of choosing only one unit from each set, two units are selected for estimation. The PRSS strategies can be implemented as follows: If the set size $$l$$ is even, $$\left( {{\raise0.7ex\hbox{${l^{2} }$} \!\mathord{\left/ {\vphantom {{l^{2} } 2}}\right.\kern-0pt} \!\lower0.7ex\hbox{$2$}}} \right)$$ units are randomly selected from the population. These units are then divided among $$\left( {{\raise0.7ex\hbox{$l$} \!\mathord{\left/ {\vphantom {l 2}}\right.\kern-0pt} \!\lower0.7ex\hbox{$2$}}} \right)$$ sets, with each set comprising $$l$$ units. The items within each set are ranked by incorporating sources, such as expert insights or auxiliary data. Subsequently, the first and $$l$$th ranked units from the initial set are chosen, followed by the second and $$\left( {l - 1} \right)th$$ units from the second set, and so on, until the $$\left( {{\raise0.7ex\hbox{$l$} \!\mathord{\left/ {\vphantom {l 2}}\right.\kern-0pt} \!\lower0.7ex\hbox{$2$}}} \right)th$$ and $$\left( {{\raise0.7ex\hbox{$l$} \!\mathord{\left/ {\vphantom {l 2}}\right.\kern-0pt} \!\lower0.7ex\hbox{$2$}} + 1} \right)th$$ elements taken from the last set. In the case of an odd value of *l*, $$\left( {{\raise0.7ex\hbox{${l\left( {l + 1} \right)}$} \!\mathord{\left/ {\vphantom {{l\left( {l + 1} \right)} 2}}\right.\kern-0pt} \!\lower0.7ex\hbox{$2$}}} \right)th$$ elements are taken directly from the under study population. The PRSS procedure involves randomly distributing the selected units among $$\left( {{\raise0.7ex\hbox{${\left( {l + 1} \right)}$} \!\mathord{\left/ {\vphantom {{\left( {l + 1} \right)} 2}}\right.\kern-0pt} \!\lower0.7ex\hbox{$2$}}} \right)th$$ sets, where each set consists of $$l$$ units. This finalizes one cycle of the PRSS procedure. The entire method can be repeated *r* times if necessary to get the desired sample size $$n = lr$$. The procedure for the PRSS can be described as follows: consider a specific cycle, denoted as *r*. within this cycle, let $$Z_{i(j),r}$$, $$i,j$$ = 1,2,3 … *l*; *r* = 1, 2, 3 … *c*, represents the jth order statistic in the *i*th sample, with cycle *r*. In this context, the RSS is used to estimate the population mean, and the estimator under PRSS approach for a single cycle is computed using the following for even *l* is given as

7 $$\overline{Z}_{{\left( {PRSS} \right)e}} = \frac{1}{l}\left[ {\sum\limits_{i = 1}^{{{\raise0.7ex\hbox{$l$} \!\mathord{\left/ {\vphantom {l 2}}\right.\kern-0pt} \!\lower0.7ex\hbox{$2$}}}} {Z_{i\left( i \right)} } + \sum\limits_{i = 1}^{{{\raise0.7ex\hbox{$l$} \!\mathord{\left/ {\vphantom {l 2}}\right.\kern-0pt} \!\lower0.7ex\hbox{$2$}}}} {Z_{{i\left( {l + 1 - i} \right)}} } } \right],$$

and

8 $${\text{var}} \left( {\overline{Z}_{{\left( {PRSS} \right)e}} } \right) = {\text{var}} \left( {\overline{Z}_{{\left( {RSS} \right)}} } \right) + \frac{2}{{l^{2} }}\sum\limits_{i = 1}^{{{\raise0.5ex\hbox{$\scriptstyle l$} \kern-0.1em/\kern-0.15em \lower0.25ex\hbox{$\scriptstyle 2$}}}} {\sum\limits_{i < l + 1 - i}^{{{\raise0.5ex\hbox{$\scriptstyle l$} \kern-0.1em/\kern-0.15em \lower0.25ex\hbox{$\scriptstyle 2$}}}} {{\text{cov}} \left( {Z_{\left( i \right)} ,Z_{{\left( {l + 1 - i} \right)}} } \right)} } .$$

For odd $$l$$

9 $$\overline{Z}_{{\left( {PRSS} \right)o}} = \frac{1}{l}\left[ {\sum\limits_{i = 1}^{{{\raise0.7ex\hbox{${\left( {l + 1} \right)}$} \!\mathord{\left/ {\vphantom {{\left( {l + 1} \right)} 2}}\right.\kern-0pt} \!\lower0.7ex\hbox{$2$}}}} {Z_{i\left( i \right)} } + \sum\limits_{i = 1}^{{{\raise0.7ex\hbox{${\left( {l - 1} \right)}$} \!\mathord{\left/ {\vphantom {{\left( {l - 1} \right)} 2}}\right.\kern-0pt} \!\lower0.7ex\hbox{$2$}}}} {Z_{{i\left( {l + 1 - i} \right)}} } } \right],$$

and variance

10 $${\text{var}} \left( {\overline{Z}_{{\left( {PRSS} \right)o}} } \right) = {\text{var}} \left( {\overline{Z}_{{\left( {RSS} \right)}} } \right) + \frac{2}{{l^{2} }}\sum\limits_{i = 1}^{{{\raise0.5ex\hbox{$\scriptstyle {\left( {l - 1} \right)}$} \kern-0.1em/\kern-0.15em \lower0.25ex\hbox{$\scriptstyle 2$}}}} {\sum\limits_{i < l + 1 - i}^{{{\raise0.5ex\hbox{$\scriptstyle {\left( {l - 1} \right)}$} \kern-0.1em/\kern-0.15em \lower0.25ex\hbox{$\scriptstyle 2$}}}} {{\text{cov}} \left( {Z_{\left( i \right)} ,Z_{{\left( {l + 1 - i} \right)}} } \right)} } .$$

### Extreme pair ranked set sampling

A modified version of the PRSS method, proposed by Balci et al.^32^ and referred to as extreme PRSS (EPRSS), introduces an innovative approach to sample selection. EPRSS is particularly valuable in cases where the population follows a heavy-tailed distribution, a scenario more common than a normal distribution. This modification addresses the limitations of standard sampling techniques, which often struggle to capture extreme values in datasets with heavy-tailed distributions. By identifying and accounting for these extreme values, EPRSS aids in producing more accurate and representative estimates, thereby mitigating potential biases that could arise from skewed estimations. The EPRSS method entails the following steps: If *l* is even, a certain number of sampling units, specified as $$\left( {{\raise0.7ex\hbox{${l^{2} }$} \!\mathord{\left/ {\vphantom {{l^{2} } 2}}\right.\kern-0pt} \!\lower0.7ex\hbox{$2$}}} \right)$$, are taken from the population concerned. These units are divided into $$\left( {{\raise0.7ex\hbox{$l$} \!\mathord{\left/ {\vphantom {l 2}}\right.\kern-0pt} \!\lower0.7ex\hbox{$2$}}} \right)$$ sets of comparable size. Following this, elements in each group are arranged in ascending sequence, and measurements are obtained from the initial and final elements in each ordered group. However, if the value of *l* is odd, an alternative method is implemented. In this case, a total of $$\left( {{\raise0.7ex\hbox{${l\left( {l + 1} \right)}$} \!\mathord{\left/ {\vphantom {{l\left( {l + 1} \right)} 2}}\right.\kern-0pt} \!\lower0.7ex\hbox{$2$}}} \right)$$ elements taken from population under study. These units are randomly distributed into $$\left( {{\raise0.7ex\hbox{${l - 1}$} \!\mathord{\left/ {\vphantom {{l - 1} 2}}\right.\kern-0pt} \!\lower0.7ex\hbox{$2$}}} \right)$$ sets, and all elements in each set are ranked accordingly. This comprehensive process enables the identification and collection of specific data points from the population, facilitating a more nuanced and inclusive analysis within the EPRSS framework. If vital, the entire EPRSS technique is recurrent *r* times to get a sample of size $$n = lr$$. The technique for estimating the mean and variance in EPRSS for a one rotation is given as follows: In case of *l* being even, the estimator is given by:

11 $$\overline{Z}_{{\left( {EPRSS} \right)e}} = \frac{1}{l}\sum\limits_{i = 1}^{{\tfrac{l}{2}}} {\left[ {Z_{i\left( 1 \right)} + Z_{i\left( l \right)} } \right]} ,$$

with variance

12 $$Var\left( {\overline{Z}_{{\left( {EPRSS} \right)e}} } \right) = \frac{1}{2l}\left[ \begin{gathered} Var\left( {Z_{\left( 1 \right)} } \right) + Var\left( {Z_{\left( l \right)} } \right) \hfill \\ + 2Cov\left( {Z_{\left( 1 \right)} ,Z_{\left( l \right)} } \right) \hfill \\ \end{gathered} \right].$$

If $l$ is odd then

13 $$\overline{Z}_{{\left( {EPRSS} \right)o}} = \frac{1}{l}\left[ {\sum\limits_{i = 1}^{{{\raise0.5ex\hbox{$\scriptstyle {\left( {l - 1} \right)}$} \kern-0.1em/\kern-0.15em \lower0.25ex\hbox{$\scriptstyle 2$}}}} {\left( {Z_{i\left( 1 \right)} + Z_{i\left( l \right)} } \right) + } Z_{{\frac{l + 1}{2}\left( {\frac{l + 1}{2}} \right)}} } \right],$$

and

14 $$Var\left( {\overline{Z}_{{\left( {EPRSS} \right)o}} } \right) = \frac{l - 1}{{2l^{2} }}\left[ \begin{gathered} Var\left( {Z_{\left( 1 \right)} } \right) + Var\left( {Z_{\left( l \right)} } \right) \hfill \\ + 2Cov\left( {Z_{\left( 1 \right)} ,Z_{\left( l \right)} } \right) \hfill \\ \end{gathered} \right] + \frac{1}{{l^{2} }}\left[ {Var\left( {Z_{{\left( {\frac{l + 1}{2}} \right)}} } \right)} \right].$$

### Quartile pair ranked set sampling

Tayyab et al.^33^ proposed the Quartile Paired RSS (QPRSS) design as an approach for estimating population parameters. The QPRSS technique can be summarized as follows: if *l* is an even number, $$\left( {{\raise0.7ex\hbox{${l^{2} }$} \!\mathord{\left/ {\vphantom {{l^{2} } 2}}\right.\kern-0pt} \!\lower0.7ex\hbox{$2$}}} \right)$$ elements are randomly drawn from the available population and allocated to $$\left( {{\raise0.7ex\hbox{$l$} \!\mathord{\left/ {\vphantom {l 2}}\right.\kern-0pt} \!\lower0.7ex\hbox{$2$}}} \right)$$ sets, with each set having a size of *l*. The elements selected within each set are ranked using cost-effective sources. Subsequently, the $$\left( {{\raise0.7ex\hbox{${\left( {l + 1} \right)}$} \!\mathord{\left/ {\vphantom {{\left( {l + 1} \right)} 4}}\right.\kern-0pt} \!\lower0.7ex\hbox{$4$}}} \right)th$$ and $$\left( {{\raise0.7ex\hbox{${3\left( {l + 1} \right)}$} \!\mathord{\left/ {\vphantom {{3\left( {l + 1} \right)} 4}}\right.\kern-0pt} \!\lower0.7ex\hbox{$4$}}} \right)th$$ ordered elements from each set are chosen. If *l* is an odd number, $$\left( {{\raise0.7ex\hbox{${l\left( {l + 1} \right)}$} \!\mathord{\left/ {\vphantom {{l\left( {l + 1} \right)} 2}}\right.\kern-0pt} \!\lower0.7ex\hbox{$2$}}} \right)$$ ordered elements are randomly taken from the population and allocated to $$\left( {{\raise0.7ex\hbox{${\left( {l + 1} \right)}$} \!\mathord{\left/ {\vphantom {{\left( {l + 1} \right)} 2}}\right.\kern-0pt} \!\lower0.7ex\hbox{$2$}}} \right)$$ sets. After ordering the units in each set, the $$\left( {{\raise0.7ex\hbox{${\left( {l + 1} \right)}$} \!\mathord{\left/ {\vphantom {{\left( {l + 1} \right)} 4}}\right.\kern-0pt} \!\lower0.7ex\hbox{$4$}}} \right)th$$ and $$\left( {{\raise0.7ex\hbox{${3\left( {l + 1} \right)}$} \!\mathord{\left/ {\vphantom {{3\left( {l + 1} \right)} 4}}\right.\kern-0pt} \!\lower0.7ex\hbox{$4$}}} \right)th$$ ordered units from the $$\left( {{\raise0.7ex\hbox{${\left( {l - 1} \right)}$} \!\mathord{\left/ {\vphantom {{\left( {l - 1} \right)} 2}}\right.\kern-0pt} \!\lower0.7ex\hbox{$2$}}} \right)$$ sets, along with the $$\left( {{\raise0.7ex\hbox{${\left( {l + 1} \right)}$} \!\mathord{\left/ {\vphantom {{\left( {l + 1} \right)} 2}}\right.\kern-0pt} \!\lower0.7ex\hbox{$2$}}} \right)th$$ unit from the enduring last set, are quantified to complete a single cycle. If essential, repeat the preceding procedures r times to obtain the needed sample size *n* = *lr*.

The mean estimator for QPRSS for a single series is specified as follows: In case *l* is an even number, mean estimator is mathematized as

15 $$\overline{Z}_{{\left( {QPRSS} \right)e}} = \frac{1}{l}\left[ {\sum\limits_{i = 1}^{{{\raise0.5ex\hbox{$\scriptstyle l$} \kern-0.1em/\kern-0.15em \lower0.25ex\hbox{$\scriptstyle 2$}}}} {Z_{{i\left( {q_{1} \left( {l + 1} \right):l} \right)}} + } \sum\limits_{i = 1}^{{{\raise0.5ex\hbox{$\scriptstyle l$} \kern-0.1em/\kern-0.15em \lower0.25ex\hbox{$\scriptstyle 2$}}}} {Z_{{i\left( {q_{3} \left( {l + 1} \right):l} \right)}} } } \right],$$

and if $l$ is odd then

16 $$\overline{Z}_{{\left( {QPRSS} \right)o}} = \frac{1}{l}\left[ \begin{gathered} \sum\limits_{i = 1}^{{{\raise0.5ex\hbox{$\scriptstyle l$} \kern-0.1em/\kern-0.15em \lower0.25ex\hbox{$\scriptstyle 2$}}}} {Z_{{i\left( {q_{1} \left( {l + 1} \right):l} \right)}} + } \sum\limits_{i = 1}^{{{\raise0.5ex\hbox{$\scriptstyle l$} \kern-0.1em/\kern-0.15em \lower0.25ex\hbox{$\scriptstyle 2$}}}} {Z_{{i\left( {q_{3} \left( {l + 1} \right):l} \right)}} } \hfill \\ + Z_{{\frac{l + 1}{2}\left( {q_{2} \left( {l + 1} \right):l} \right)}} \hfill \\ \end{gathered} \right],$$

respective variances are

17 $$Var\left( {\overline{Z}_{{\left( {QPRSS} \right)e}} } \right) = \frac{1}{2l}\left[ \begin{gathered} \delta_{{\left( {q_{1} \left( {l + 1} \right)} \right)}}^{2} + \delta_{{\left( {q_{3} \left( {l + 1} \right)} \right)}}^{2} \hfill \\ + 2\delta_{{\left( {q_{1} \left( {l + 1} \right),q_{3} \left( {l + 1} \right)} \right)}} \hfill \\ \end{gathered} \right],$$

and

18 $$Var\left( {\overline{Z}_{{\left( {QPRSS} \right)o}} } \right) = \frac{l - 1}{{2l^{2} }}\left[ \begin{gathered} \delta_{{\left( {q_{1} \left( {l + 1} \right)} \right)}}^{2} + \delta_{{\left( {q_{3} \left( {l + 1} \right)} \right)}}^{2} \hfill \\ + 2\delta_{{\left( {q_{1} \left( {l + 1} \right),q_{3} \left( {l + 1} \right)} \right)}} \hfill \\ \end{gathered} \right] + \frac{1}{{l^{2} }}\delta_{{\left( {q_{1} \left( {l + 1} \right)} \right)}}^{2} .$$

## Suggested AEWMA CC applying with various PRSS schemes utilizing Bayesian methodology

The section discusses the recommended CC applying distinct PRSS strategies for monitoring the process parameter of a normally distribution. Consider the independently and identically normally distributed variable *i.e.,* $X_{1} ,X_{2} ,...X_{n}$ with θ and $\sigma^{2}$ respectively. As a result, the probability function can be expressed as:

19 $$f\left( {x_{t} :\theta ,\sigma^{2} } \right) = \frac{1}{{\sqrt {2\pi \sigma^{2} } }}\exp \left( { - \tfrac{1}{{2\sigma^{2} }}\left( {x_{t} - \theta } \right)^{2} } \right).$$

The estimation of the mean shift is denoted by ${\widehat{\delta }}_{t}^{*}$ and is considered as the sequence of EMWA statistics using $\left\{ {X_{t} } \right\}$. Mathematically, this is expressed as

20 $$\hat{\delta }_{t}^{*} = \psi X_{t} + \left( {1 - \psi } \right)\hat{\delta }_{t - 1}^{*} ,$$

where $\hat{\delta }_{0}^{*} = 0$ and $\psi$ denotes the smoothing constant, the estimator $\hat{\delta }_{0}^{*}$ exhibits bias for out-of-control processes and is unbiased for in-control processes. Haq et al.^9^ have contributed an equitable approximation of σ that is suitable for both processes within control and those that are out of control. This impartial estimation can be mathematically articulated as:

21 $$\hat{\delta }_{t}^{**} = \frac{{\hat{\delta }_{t}^{*} }}{{1 - \left( {1 - \psi } \right)^{t} }}.$$

It is offered to use $$\hat{\delta }_{t} = \left| {\hat{\delta }_{t}^{**} } \right|$$ for $$\delta$$ estimation.

The proposed statistic applying PRSS designs utilizing Bayesian technique for estimating the process mean using the sequence $\left\{ {X_{t} } \right\}$ is provided by:

22 $$F_{t} = g\left( {\hat{\delta }_{t} } \right)\hat{\theta }_{{\left( {RSS_{i} } \right)LF}} \, + \left( {1 - g\left( {\hat{\delta }_{t} } \right)} \right)F_{t - 1} ,$$

where $$i = 1,2,3,$$$$RSS_{1} = PRSS$$, $$RSS_{2} = QPRSS$$, $$RSS_{3} = EPRSS$$,$$g\left( {\hat{\delta }_{t} } \right) \in \left( {0,\left. 1 \right]} \right.$$ and $$F_{0} = 0$$ such that g

23 $$\left({\widehat{\delta }}_{t}\right)=\left\{\begin{array}{*{20}l}\frac{1}{a\left[1+{\left({\widehat{\delta }}_{t}\right)}^{-c}\right]}& \quad if \,0<{\widehat{\delta }}_{t}\le 2.7\\ 1 & \quad if \,{\widehat{\delta }}_{t}>2.7\end{array}\right..$$

Atif et al.^34^ presented a function, labeled as (23), aimed at adjusting the smoothing constant while factoring shift estimated. The recommended constants utilized in $g\left( {\hat{\delta }_{t} } \right)$ is $a = 7$ and $c = 1$, when $1 < \hat{\delta }_{t} \le 2.7$, the value of $c = 2$ for $\hat{\delta }_{t} \le 1$. When the Bayesian AEWMA plotting statistic exceeds the predetermined threshold value h, it signals an out-of-control process. And if the statistic remains lower than the assigned threshold value, it signifies a process under control.

In scenarios where probability function and the prior distribution adhere to a normal distribution, the subsquent posterior distribution also conforms to a normal distribution, characterized by θ_n_ and σ^2^_n,_ mean and variance respectively. The $P\left( {\theta /x} \right)$ can be mathematically represented as:

24 $$P\left( {\theta /x} \right) = \frac{1}{{\sqrt {2\pi } \sqrt {\frac{{\delta^{2} \delta_{0}^{2} }}{{\delta^{2} + n\delta_{0}^{2} }}} }}*\exp \left[ { - \frac{1}{2}\left( {\frac{{\theta - \sum\limits_{i = 1}^{n} {\frac{{x_{i} \delta_{0}^{2} + \theta_{0} \delta_{0}^{2} }}{{\delta^{2} + n\delta_{0}^{2} }}} }}{{\sqrt {\frac{{\delta^{2} \delta_{0}^{2} }}{{\delta^{2} + n\delta_{0}^{2} }}} }}} \right)} \right],$$

where, $$\theta_{n} = \frac{{n\overline{x} \delta_{0}^{2} + \delta^{2} \theta_{0} }}{{\delta^{2} + n\delta_{0}^{2} }}$$ and $$\delta_{n}^{2} = \frac{{\delta^{2} \delta_{0}^{2} }}{{\delta^{2} + n\delta_{0}^{2} }}$$.

The estimator for the suggested approach, which incorporates the Bayesian methodology under various PRSS designs utilizes the SELF, can be expressed as follows:

The estimator for the offered approach, which integrates Bayesian methodology across various PRSS designs employs the SELF, is mathematically represented as follows:

25 $$\hat{\theta }_{{\left( {SELF} \right)}} = \frac{{n\overline{x}_{{(PRSS_{i} )}} \delta_{0}^{2} + \delta^{2} \theta_{0} }}{{\delta^{2} + n\delta_{0}^{2} }}.$$

The properties of the $\hat{\theta }_{{_{{\left( {SELF} \right)}} }}$ is expressed, $E\left( {\hat{\theta }_{{\left( {SELF} \right)}} } \right) = \frac{{n\theta_{1} \delta_{0}^{2} + \delta^{2} \theta_{0} }}{{\delta^{2} + n\delta_{0}^{2} }}$ and $sd\left( {\hat{\theta }_{{\left( {SELF} \right)}} } \right) = \sqrt {\frac{{n\delta_{{(PRSS_{i} )}}^{2} \delta_{0}^{4} }}{{\delta^{2} + n\delta_{0}^{2} }}}$. The Bayes estimator utilizing the LLF and with PRSS, can be calculated as follows:

26 $$\hat{\theta }_{{\left( {_{LLF} } \right)}} = \frac{{n\overline{x}_{{(PRSS_{i} )}} \delta_{0}^{2} + \delta^{2} \theta_{0} }}{{\delta^{2} + n\delta_{0}^{2} }} - \frac{{C{\prime} }}{2}\delta_{n}^{2} .$$

The mean of $\hat{\theta }_{{\left( {LLF} \right)}}$ is mathematized as $E\left( {\hat{\theta }_{LLF} } \right) = \frac{{n\theta_{1} \delta_{0}^{2} + \delta^{2} \theta_{0} }}{{\delta^{2} + n\delta_{0}^{2} }} - \frac{{C{\prime} }}{2}$.

Suppose there are future observations of size *h*, denoted as y_1_, y_2_, …, y_n_. In context of Bayesian methodology, employing different RSS strategies for posterior predictive distribution, the P(y/x) can be represented as:

27 $$p\left( {{\raise0.7ex\hbox{$y$} \!\mathord{\left/ {\vphantom {y x}}\right.\kern-0pt} \!\lower0.7ex\hbox{$x$}}} \right) = \frac{1}{{\sqrt {2\pi \delta_{1}^{2} } }}\exp \left\{ { - \frac{1}{{2\delta_{1}^{2} }}\left( {Y - \theta_{n} } \right)^{2} } \right\},$$

where $${\raise0.7ex\hbox{$y$} \!\mathord{\left/ {\vphantom {y x}}\right.\kern-0pt} \!\lower0.7ex\hbox{$x$}}$$ is normally distributed having mean $$\theta_{n}$$ and the standard deviation $$\delta_{1}$$, mathematized as $$\delta_{1} = \sqrt {\delta^{2} + \frac{{\delta^{2} \delta_{0}^{2} }}{{\delta^{2} + n\delta_{0}^{2} }}}$$. Then $$\theta$$ is estimated for posterior predictive distribution applying LLF with different PRSS designs by

28 $$\hat{\theta }_{LLF} = \frac{{n\overline{x}_{{(PRSS_{i} )}} \delta_{0}^{2} + \delta^{2} \theta_{0} }}{{\delta^{2} + n\delta_{0}^{2} }} - \frac{{C^{\prime } }}{2}\tilde{\delta }_{1}^{2} ,$$

where, $\tilde{\delta }_{1}^{2} = \frac{{\delta^{2} }}{k} + \frac{{\delta^{2} \delta_{0}^{2} }}{{\delta^{2} + n\delta_{0}^{2} }}$, $E\left( {\hat{\theta }_{LLF} } \right) = \frac{{n\theta_{1} \delta_{0}^{2} + \delta^{2} \theta_{0} }}{{\delta^{2} + n\delta_{0}^{2} }} - \frac{{C{\prime} }}{2}\tilde{\delta }_{1}^{2}$ and $sd\left( {\hat{\theta }_{LLF} } \right) = \sqrt {\frac{{n\delta_{{(PRSS_{i} )}}^{2} \delta_{0}^{4} }}{{\left( {\delta^{2} + n\delta_{0}^{2} } \right)^{2} }}}$ are the mean and standard deviation of $\hat{\theta }_{LLF}$.

## Simulation study

The effectiveness of the AEWMA CC, which incorporates Bayesian methodology and is applicable to various PRSS designs, is evaluated using Monte Carlo simulation. The evaluation process encompasses various measures, including the ARL and the SDRL. To evaluate the impact of the proposed CC with different LFs, smoothing constants of $\psi$ = 0.10 and 0.25 are utilized. The state of an in-control process is indicated at 370. Hereafter, we present a summary of the essential simulation steps required to implement the offered CC.

**Step 1**: Setting in-control ARL

- The prior and sampling distribution are assumed to follow a standard normal distribution, from which the properties are determined for different LFs. *i.e.,* $E\left( {\hat{\theta }_{{\left( {_{LLF} } \right)}} } \right)$ and $\delta_{LLF}$.
- The determination of the threshold value '*h*' is grounded on a particular chosen smoothing constant value.
- For an in-control process, generate a random samples from a normal distribution of size n, $X \sim N\left( {E\left( {\hat{\theta }} \right),\delta^{2} } \right)$.
- Compute the recommended AEWMA statistic and assess the process in line with the predetermined design specifications.
- Repeat the preceding three stages indefinitely as long as the process stays under control, and maintain track of the number of run lengths for the under-control process until it is identified as out-of-control.

**Step 2**: For out-of-control ARL

- In the case of a shifted process, draw samples from a Gaussian distribution. i.e., $X \sim N\left( {E\left( {\hat{\theta }_{{_{LF} }} } \right) + \sigma \frac{\delta }{\sqrt n },\delta } \right)$.
- Compute the statistic Ft for the AEWMA using a Bayesian approach, and assess the process under the offered design.
- Continue to repeat the above-mentioned steps as long as the process remains within control, while keeping a record of the run length for the in-control process.
- Perform iterations of steps (i–iii) for a total of 100,000 times, and compute the ARL and SDRL.

## Results and discussions

Tables 1, 2, 3, 4, 5 and 6 present a detailed comparison between the proposed methodology and the existing chart that employs Bayesian approach using SRS. The suggested CC is developed through the implementation of distinct PRSS strategies, each utilizing two distinct LFs. The observations suggest that the suggested CC demonstrates a more pronounced ability to effectively monitor the mean of the process when compared with available Bayesian chart that utilizes SRS based on the analysis of performance measures such as the ARL and the SDRL values of the offered CC, which are derived from the PRSS schemes utilizing the SELF under an informative prior. This performance is notably superior to that of the Bayesian AEWMA CC, which employs SRS. As an illustration, consider the results obtained from the available Bayesian chart applying SRS with a specific $\psi$ = 0.10. The ARL values for distinct shifts, such as 0.0, 0.30, 0.50, 0.80, 1.50, and 4, are 370.16, 43.59, 18.90, 7.90, 2.56, and 1.01, respectively. In a similar scenario, the *ARL* values of offered CC, employing PRSS are 370.51, 23.55, 9.20, 3.73, 1.45, and 1, while those under QPRSS are 369.17, 22.46, 8.73, 3.55, 1.40, and 1. Furthermore, the run length outcomes of the offered CC under EPRSS are 371.14, 24.20, 9.52, 3.94, 1.49, and 1. As an illustration, consider the results obtained from the existing CC using SRS, with SELF under $\psi$ = 0.10. ARL values for various shifts, such as 0.0, 0.30, 0.50, 0.80, 1.50, and 4, are 370.16, 43.59, 18.90, 7.90, 2.56, and 1.01, respectively. In a similar scenario, the ARL values for the offered chart, employing PRSS, are 370.51, 23.55, 9.20, 3.73, 1.45, and 1, while those under QPRSS are 369.17, 22.46, 8.73, 3.55, 1.40, and 1. Furthermore, ARL output of recommended chart utilizing EPRSS are 371.14, 24.20, 9.52, 3.94, 1.49, and 1. The findings illustrate the effectiveness of the offered chart when applying PRSS designs. Additionally, a comparison is made between the effectiveness of the Bayesian chart applying SRS and the suggested CC under PRSS methods, which include an informative prior and two distinct LFs at $\psi$ = 0.25. These comparisons are conducted across different shift values such as 0.0, 0.30, 0.50, 0.80, 1.50, and 4, revealing ARLs of 369.50, 55.71, 27.40, 12.96, 4.08, and 1.08, respectively. ARL outcomes of recommended method utilizing PRSS demonstrate values of 371.18, 28.96, 14.91, 6.24, 2.02, and 1 for various shift magnitudes. In contrast, employing QPRSS yields ARL output are 370.56, 31.82, 14.12, 5.83, 1.93, and 1. Furthermore, when utilizing EPRSS, the ARL outputs are 369.23, 21.93, 11.03, 6.41, 1.37, and 1 for shifts of differing magnitudes. In contrast to AEWMA chart, which uses Bayesian approach under SRS, the results suggest that the proposed methodology shows a rapid decay in values ​​under PRSS systems, especially at larger shifts. This bearish trend is a testament to the system's superior ability to effectively detect runaway signals within the monitored process. These findings can be summarized briefly and succinctly in the following key points.

**Figure 1:**
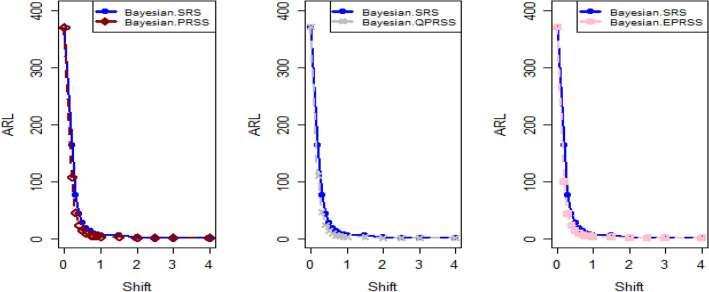
Using SELF, Plots for P and PP distribution.

**Figure 2:**
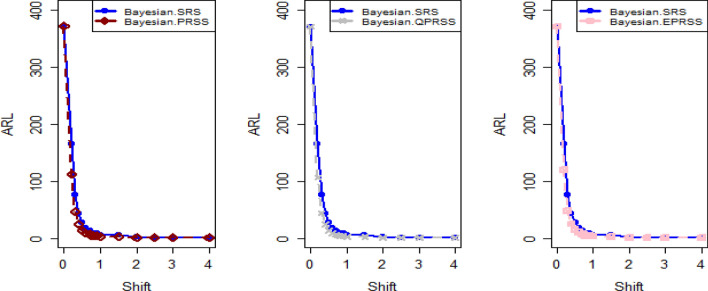
ARL graphs for P distribution applying LLF with PRSS designs.

**Figure 3:**
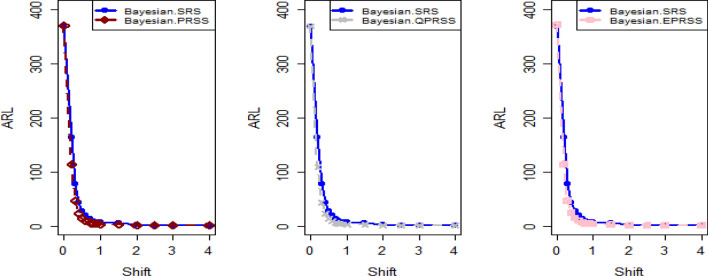
Graphs for PP distribution with LLF using PRSS schemes.

**Figure 4:**
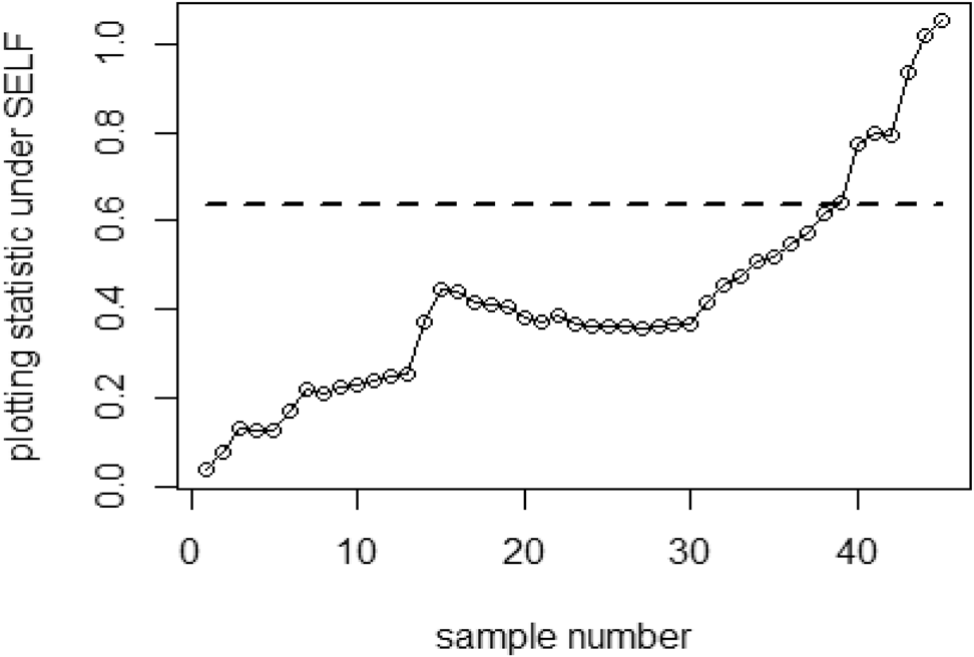
Based on SRS, the ARL graph for the Bayesian chart with SELF.

**Figure 5:**
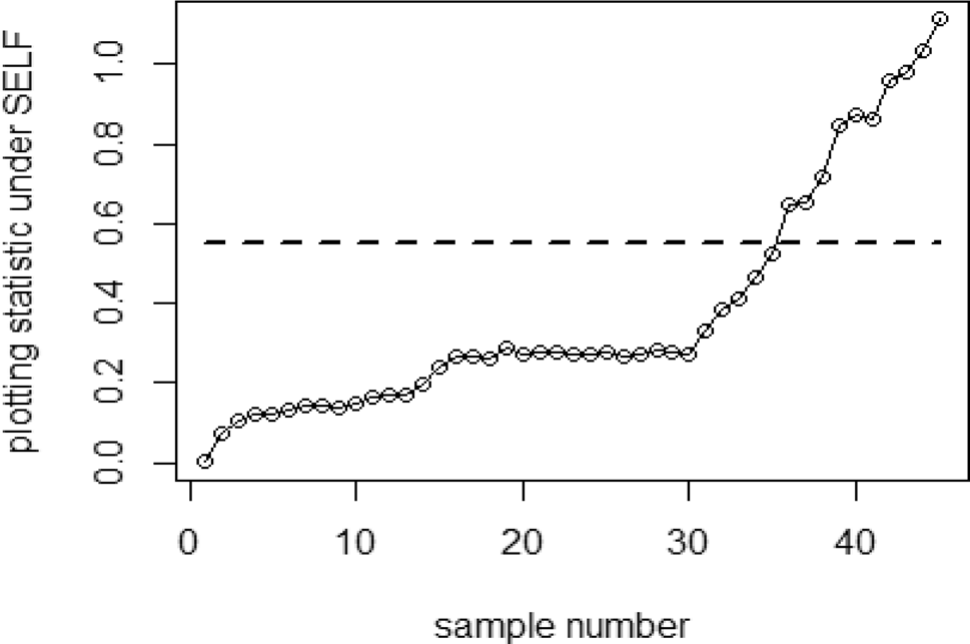
Using PRSS, ARL graph for Bayesian CC under SELF.

**Figure 6:**
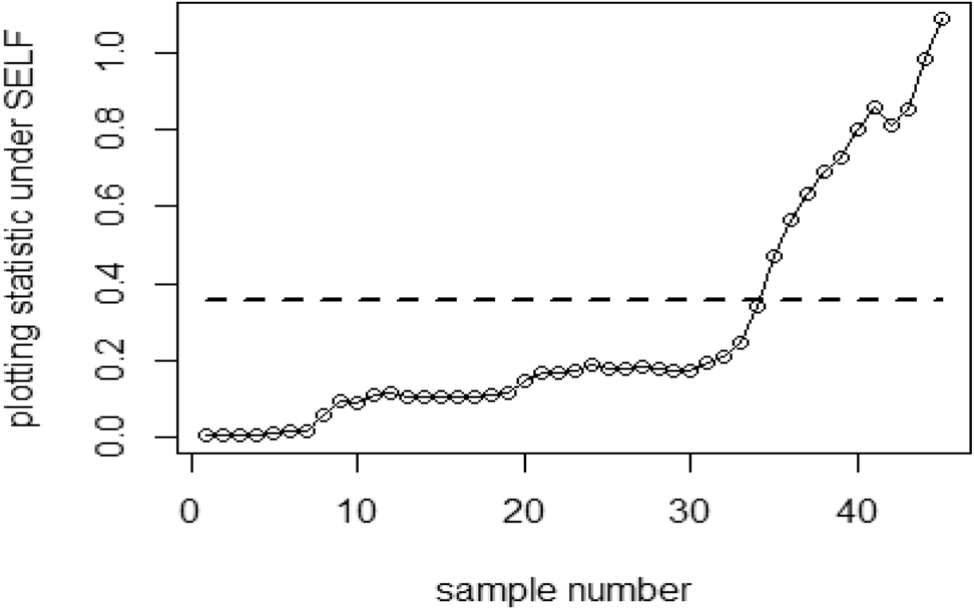
Using QPRSS, the graph shows Bayesian AEWMA CC based on SELF.

**Figure 7:**
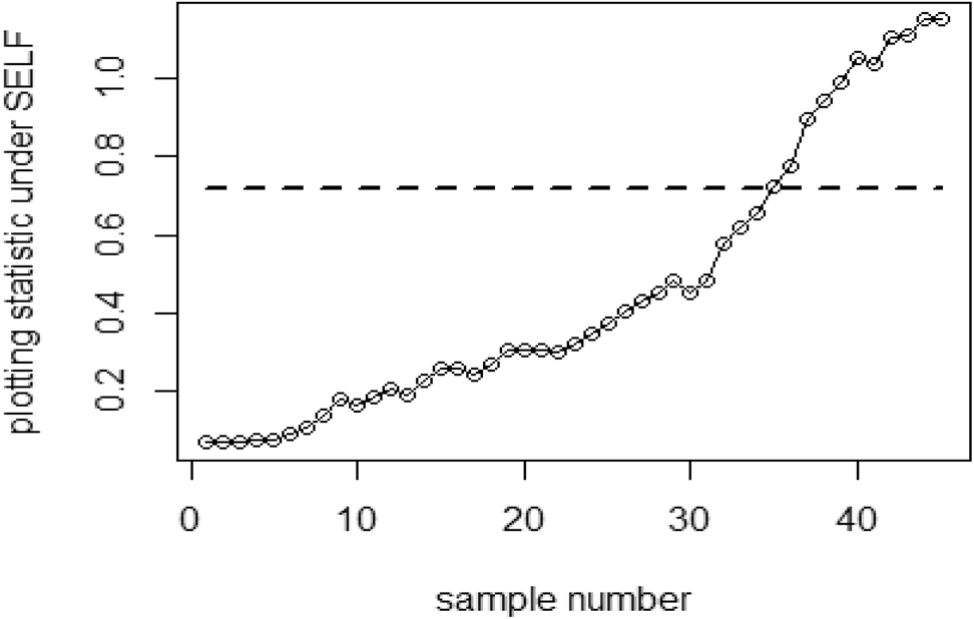
Using EPRSS, graph shows AEWMA chart utilizing SELF.

- Analysis of the ARL results for the proposed CC applying SELF across distinct PRSS designs shows a consistent and rapid decrease in values ​​with increasing shift in the process mean. This trend designates that the offered technique remains unbiased, as shown in Tables 1 and 2. For example, looking at the results in Table 1 with an ARL = 370 and a smoothing constant (δ) set to various shifts such as 0.20 and 0.70, the ARL values ​​are 44.39 and 4 for PRSS 0.70, for QPRSS at 46.01 and 4.77 and for EPRSS at 42.81 and 4.78.
- From Tables 3 and 4, it can be seen that the performance of the offered technique is susceptible to variations in the value of $\psi$, which are given as 0.10 and 0.25. Considering the LLF, ARL and SDRL outcomes for the offered method with emphasis on the P distribution are presented in Tables 3 and 4. These tables illustrate a decrease in efficiency as the smoothing constant increases for offered chart. For example, with ARL = 370 and $\psi$ = 0.10 along with a shift of 0.20, respective ARL outputs of suggested chart using PRSS, QPRSS, and EPRSS are 46.17, 44.18, and 47.89, respectively. Furthermore, the ARL values ​​for the same displacement (δ) of 0.20 are 54.76 for PRSS, 52.23 for QPRSS, and 56.82 for EPRSS, respectively.
- The run length output of the offered chart under various PRSS schemes are presented in Tables 5 and 6. These tables offer valuable information on how the proposed chart performs when employing PRSS methodologies with the LLF. Specifically, at ARL = 370, with a shift (δ) value of 0.50 and a smoothing constant (sci) set at 0.10, the ARL value is 9.35. Similarly, when the smoothing constant (sci) is set to 0.25, the ARL value is 14.81. Comparatively, for the same scenario, the ARL values using QPRSS are 9.05 and 13.00, while those obtained using EPRSS are 9.90 and 14.90, respectively.
- From An examination of Tables 1, 2, 3, 4, 5 and 6 reveals that the suggested chart displays a relatively higher susceptibility in identifying out-of-control with the comparison to the Bayesian CC that utilizes SRS. This decision is drawn from the Figs. 1, 2, 3, 4, 5, 6 and 7, which provides clear evidence of the offered Bayesian AEWMA CC comparatively limited effectiveness in identifying deviations from the expected process behavior. The r codes for the proposed design are included in the Appendix A.

**Table 1:** ARL and SDRL outcomes with SELF for proposed CC based on Bayesian theory, for $\psi$ = 0.10, *n* = *5.*

Shift	Baye-SRS		Baye-PRSS		Baye-QPRSS		Baye-EPRSS	
	ARL	SDRL	ARL	SDRL	ARL	SDRL	ARL	SDRL
	h = 0.0311		h = 0.0103		h = 0.0101		h = 0.00910	
0.00	370.16	432.35	370.07	449.33	371.55	430.42	370.66	538.77
0.10	162.84	163.19	107.98	105.50	110.53	102.85	99.54	101.76
0.20	75.68	71.49	44.39	42.27	46.01	42.29	42.81	41.66
0.30	43.59	40.55	23.39	22.55	23.79	22.24	22.55	22.16
0.40	27.70	25.99	13.97	13.62	13.98	13.17	13.61	13.47
0.50	18.90	17.94	9.12	8.73	9.08	8.41	8.87	8.61
0.60	13.88	13.22	6.43	5.98	6.20	5.68	6.37	5.94
0.70	10.22	9.48	4.70	4.18	4.77	4.20	4.78	4.35
0.75	9.07	8.33	4.21	3.69	3.67	3.07	4.16	3.72
0.80	7.90	7.32	3.72	3.16	3.00	2.40	3.78	3.27
0.90	6.37	5.78	3.07	2.50	3.05	2.47	3.10	2.58
1.00	5.22	4.64	2.55	1.93	2.53	1.91	2.54	1.96
1.50	2.56	1.91	1.45	0.77	1.42	0.74	1.46	0.80
2.00	1.68	1.00	1.12	0.35	1.11	0.33	1.13	0.37
2.50	1.31	0.59	1.02	0.14	1	0	1.02	0.15
3.00	1.13	0.36	1	0	1	0	1	0
4.00	1.01	0.12	1	0	1	0	1	0

**Table 2:** Run length output of the CC when implementing SESL within the recommended CC., for $\psi$ = 0.25, *n* = *5.*

Shift	Baye-SRS		Baye-PRSS		Baye-QPRSS		Baye-EPRSS	
	ARL	SDRL	ARL	SDRL	ARL	SDRL	ARL	SDRL
	h = 0.0676		h = 0.0241		h = 0.0228		h = 0.00246	
0.00	372.48	352.52	369.16	355.16	369.41	336.54	369.41	333.74
0.10	183.06	148.18	129.87	94.08	128.24	94.03	125.37	91.64
0.20	90.14	62.61	58.17	38.29	47.39	38.31	46.43	34.52
0.30	55.36	36.35	33.84	22.13	33.24	21.37	33.72	22.31
0.40	37.71	24.29	21.91	14.72	21.11	13.94	21.05	19.56
0.50	27.45	17.76	14.95	10.22	14.21	9.61	14.87	10.33
0.60	20.67	13.59	10.79	7.51	10.32	7.06	10.82	7.61
0.70	16.22	10.76	8.17	5.69	7.63	5.37	8.15	5.68
0.75	14.28	9.65	7.07	4.97	6.76	4.78	7.24	5.08
0.80	12.91	8.64	6.31	4.35	5.96	4.13	6.25	4.36
0.90	10.37	6.94	5.03	3.46	4.79	3.29	5.02	3.53
1.00	8.69	5.83	4.10	2.77	3.91	2.65	4.22	2.86
1.50	4.13	2.63	2.02	1.15	1.94	1.09	2.08	1.22
2.00	2.52	1.44	1.36	0.60	1.32	0.57	1.37	0.62
2.50	1.78	0.91	1.10	0.31	1.08	0.29	1.12	0.34
3.00	1.41	0.62	1.02	0.14	1	0	1.02	0.16
4.00	1.08	0.27	1	0	1	0	1	0

**Table 3:** Run length outcomes by using LLF for suggested AEWMA CC, for $\psi$ = 0.10, *n* = *5.*

Shift	Baye-SRS		Baye-PRSS		Baye-QPRSS		Baye-EPRSS	
	ARL	SDRL	ARL	SDRL	ARL	SDRL	ARL	SDRL
	h = 0.086		h = 0.0105		h = 0.0102		h = 0.00116	
0.00	370.23	430.46	371.06	444.81	370.54	482.98	370.68	465.56
0.10	165.20	166.08	111.71	104.20	106.42	89.79	119.54	108.36
0.20	76.22	70.58	46.17	42.81	44.18	42.17	47.89	44.41
0.30	43.74	41.01	24.28	23.13	24.08	22.47	25.43	23.84
0.40	27.96	26.05	14.01	13.36	13.90	13.20	15.09	14.38
0.50	18.78	18.07	9.35	8.85	9.05	8.50	9.90	9.26
0.60	13.78	13.04	6.66	6.19	6.42	5.92	7.07	6.50
0.70	10.34	9.69	4.89	4.40	4.76	4.25	5.22	4.67
0.75	8.96	8.36	4.35	3.77	4.22	3.62	4.64	4.04
0.80	8.07	7.52	3.79	3.26	3.72	3.12	4.01	3.41
0.90	6.38	5.77	3.07	2.46	3.01	2.39	3.26	2.69
1.00	5.29	4.67	2.56	1.96	2.50	1.89	2.78	2.16
1.50	2.59	1.91	1.45	0.76	1.43	0.75	1.50	0.82
2.00	1.69	1.01	1.12	0.35	1.11	0.34	1.14	0.40
2.50	1.31	0.60	1.02	0.16	1.02	0.15	1.03	0.18
3.00	1.13	0.36	1	0	1	0	1	0
4.00	1.01	0.12	1	0	1	0	1	0

**Table 4:** ARLs and SDRLs values for the Bayesian AEWMA CC for P distribution applying LLF with $\psi$ = 0.25 and *n* = *5.*

Shift	Baye-SRS		Baye-PRSS		Baye-QPRSS		Baye-EPRSS	
	ARL	SDRL	ARL	SDRL	ARL	SDRL	ARL	SDRL
	h = 0.0677		h = 0.0235		h = 0.0227		h = 0.0247	
0.00	370.51	354.19	372.11	329.04	371.07	357.90	369.68	345.85
0.10	183.18	148.72	128.42	93.58	130.33	90.84	126.62	95.82
0.20	90.99	62.57	54.76	37.26	52.23	37.58	56.82	37.88
0.30	55.68	35.92	33.03	21.54	33.08	21.23	33.79	22.44
0.40	37.99	24.51	21.40	14.43	20.83	13.90	21.97	14.63
0.50	27.77	17.98	14.81	10.15	13.00	15.73	14.90	10.29
0.60	20.84	13.64	10.55	7.43	10.36	7.21	10.81	7.71
0.70	16.20	10.71	7.87	5.52	7.70	5.37	8.16	5.76
0.75	14.47	9.71	6.95	4.94	6.85	4.74	7.25	5.07
0.80	12.78	8.59	6.14	4.35	5.99	4.15	6.30	4.47
0.90	10.50	7.08	4.91	3.38	4.82	3.32	5.04	3.53
1.00	8.60	5.81	3.99	2.72	3.92	2.58	4.18	2.85
1.50	4.09	2.63	2.01	1.16	1.94	1.08	2.07	1.21
2.00	1.79	0.91	1.33	0.58	1.33	0.57	1.38	0.62
2.50	2.53	1.45	1.10	0.32	1.08	0.28	1.11	0.34
3.00	1.41	0.61	1	0	1	0	1.02	0.15
4.00	1.08	0.27	1	0	1	0	1	0

**Table 5:** Using LLF, the output for CC at $\psi$ = 0.10, *n* = *5.*

Shift	Baye-SRS		Baye-PRSS		Baye-QPRSS		Baye-EPRSS	
	ARL	SDRL	ARL	SDRL	ARL	SDRL	ARL	SDRL
	h = 0.0313		h = 0.0104		h = 0.00961		h = 0.00919	
0.00	368.52	428.40	370.51	452.05	369.17	424.92	371.14	460.30
0.10	163.25	163.46	113.29	110.83	108.86	102.96	113.48	111.05
0.20	77.39	70.69	45.71	42.83	42.90	40.32	46.61	44.05
0.30	43.89	40.84	23.55	22.62	22.46	21.71	24.20	23.25
0.40	27.89	26.26	14.20	13.68	13.25	12.91	14.42	13.87
0.50	19.04	17.98	9.20	8.73	8.73	8.34	9.52	9.13
0.60	13.60	12.86	6.46	5.96	6.20	5.74	6.65	6.28
0.70	10.33	9.75	4.91	4.37	4.50	3.95	4.95	4.50
0.75	9.00	8.43	4.32	3.78	4.03	3.49	4.45	3.94
0.80	7.97	7.34	3.73	3.16	3.55	2.96	3.94	3.42
0.90	6.48	5.88	3.09	2.52	2.96	2.40	3.17	2.55
1.00	5.25	4.61	2.58	1.96	2.47	1.86	2.66	2.09
1.50	2.57	1.90	1.45	0.78	1.40	0.73	1.49	0.81
2.00	1.71	1.02	1.11	0.35	1.10	0.33	1.14	0.39
2.50	1.31	0.59	1.02	0.16	1.02	0.14	1.03	0.17
3.00	1.13	0.37	1	0	1	0	1	0
4.00	1.01	0.12	1	0	1	0	1	0

**Table 6:** Run length results for offered CC using LLFs, at $\psi$ = 0.25, *n* = *5.*

Shift	Baye-SRS		Baye-PRSS		Baye-QPRSS		Baye-EPRSS	
	ARL	SDRL	ARL	SDRL	ARL	SDRL	ARL	SDRL
	h = 0.0674		h = 0.0238		h = 0.0127		h = 0.0212	
0.00	369.25	351.98	371.26	338.06	373.63	337.12	369.54	360.66
0.10	182.80	151.23	129.81	96.01	123.46	91.13	91.33	98.51
0.20	90.69	63.05	56.72	36.79	54.01	36.27	51.42	58.79
0.30	55.67	36.16	28.96	20.38	31.82	21.09	21.93	14.73
0.40	37.69	24.34	21.48	14.29	20.39	13.67	15.03	10.36
0.50	27.50	17.83	14.91	10.17	14.12	9.74	11.03	7.77
0.60	20.73	13.67	10.53	7.38	10.04	6.95	10.86	7.53
0.70	16.04	10.63	7.91	5.60	7.57	5.33	8.18	5.78
0.75	14.31	9.58	7.06	4.98	6.58	4.63	7.23	5.06
0.80	12.91	8.69	6.24	4.36	5.83	4.14	6.41	4.47
0.90	10.34	7.03	4.97	3.37	4.71	3.22	5.09	3.52
1.00	8.58	5.82	4.08	2.77	3.84	2.57	4.21	2.88
1.50	4.08	2.63	2.02	1.16	1.93	1.11	2.07	1.21
2.00	2.52	1.46	1.36	0.61	1.30	0.55	1.37	0.61
2.50	1.79	0.90	1.10	0.32	1.08	0.28	1.12	0.34
3.00	1.40	0.61	1.02	0.14	1	0	1	0
4.00	1.08	0.27	1	0	1	0	1	0

## Real data applications

In the realm of research, the utilization of real datasets and simulated examples is a standard practice aimed at illustrating the practical application and effectiveness of proposed charts. In the context of this particular study, a real dataset is used to showcase the operational dynamics and the practical utility of the charts. The investigation focuses on semiconductor manufacturing, particularly the integration of the hard-bake process with photolithography. The central objective revolves around establishing statistical control over the resist flow width within this process, employing both the existing and recommended chart. To achieve this, a dataset obtained from Montgomery^35^ is employed, comprising forty-five samples, each involving 5 wafers derived from the manufacturing process. These samples are taken at hourly intervals, with the measurements of flow width recorded in microns. The initial 30 samples are presumed to reflect data from an in-control process, constituting the phase 1 dataset, while the subsequent 15 samples represent data from an out-of-control process, forming the phase 2 dataset. for both the P and PP distributions.

Figure 4 depicts the application of Bayesian chart under SRS, identifying an out-of-control signal on the 40th sample. This method utilizes the SELF for P distributions, employing SRS. In contrast, Figs. 5, 6, 7 demonstrate the usage of the recommended chart that integrating P distribution applying SELF and different stratigies for the PRSS. According to the figures, the suggested CC detects out-of-control signals for PRSS, QPRSS, and EPRSS on the 36th, 33rd, and 35th samples, respectively. In summary, Figs. 1, 2, 3, 4, 5, 6 and 7 collectively emphasize the increased sensitivity of the suggested CC in detecting out-of-control signals.

## Conclusion

The implementation of the recommended CC applying PRSS schemes for both P and PP distribution has been proposed to effectively monitor process mean. This innovative methodology is meticulously compared to the availiable CC under SRS, and the comprehensive analysis is documented in Tables 1, 2, 3, 4, 5 and 6. Notably, the results obtained from the recommended approach demonstrate a superior performance compared to the conventional CC. To exemplify the practical implementation of the proposed technique, a real-world dataset is utilized, showcasing its efficacy in precisely tracking the location parameter and promptly identifying any deviations from the desired target. In order to further enhance the Bayesian AEWMA CC, the study suggests several promising research avenues. These research avenues involve delving into the method's adaptability and resilience when dealing with non-normal distributions. Additionally, they encompass an examination of alternative sampling techniques, such as consecutive sampling, to improve the precision of the control chart. By focusing on these areas of investigation, the proposed methodology can be customized to various scenarios, ultimately bolstering its efficacy in overseeing processes and ensuring quality control. This research highlights the importance of these developments in managing varied datasets and provides valuable guidance for future studies, thereby making ongoing contributions to the enhancement of process monitoring and quality management practices.

### Supplementary Information

Supplementary Information.

## Data Availability

Should anyone make a reasonable request, they can directly access the datasets used or analyzed in this study from the corresponding author.
