# Supplementary material for: Monitoring of semiconductor manufacturing process on Bayesian AEWMA control chart under paired ranked set sampling schemes
Source: Sci Rep. 2023 Dec 19;13:22703. doi: 10.1038/s41598-023-49843-2 (PMC10733340; doi:10.1038/s41598-023-49843-2)
Supplement: Supplementary file 1 — Supplementary Information. [file 41598_2023_49843_MOESM1_ESM.docx]

**Appendix A**

**R codes for the proposed design**

#ARL using posterior and predictive posterior dis under SELF

#correct version of ARL using posterior and predictive posterior dis under SELF

rm(list=ls(all=TRUE))

library(MASS)

n=m=5;

est_rss=c();rssx=matrix(,m,m);rssy=matrix(,m,m);

Mx=c(); Zp=c(); ucl=c(); lcl=c(); rl=c();

mu=0; sig=1;

rssx=matrix(,m,m);

# ----------For Standard Deviation-----------

for(i in 1:100000)

{

for(k in 1:m)

{

x=rnorm(m,0,1)

dat=data.frame(x)

xs=dat[order(x),]

rssx[k,]=xs

}

rssx_samp=diag(rssx)

Mx[i]=mean(rssx_samp)

}

sigM= sd(Mx)

sigM

Wp=c();sci=c();

ld=0.25; h=0.0546; shift=0.60;sigmp=c(); sigmt=c(); a = 7;

# Baysian Part

m_pr=0;sd_pr=1

m_po=0;sd_po=1

# Under Self

NU=(n*m_po*(sd_pr^2)+(sd_po^2)*m_pr)

DE=sd_po^2+n*sd_pr^2

muM=muW=NU/DE

sig=1

sigma=(n*sd_po^2*(sd_pr)^4)/DE^2

shi=mu+shift*sqrt(sig/n);

for(j in 1:10000)

{

for(i in 1:100000)

{

for(k in 1:m)

{

x=rnorm(m,shi,1)

dat=data.frame(x)

xs=dat[order(x),]

rssx[k,]=xs

}

y=mean(diag(rssx))

NUs=(n*mean(y)*(sd_pr)+(sd_po)*m_pr)

DEs=sd_po+n*sd_pr

Mx[i]=NUs/DEs

if(i==1)

{Zp[i]=ld*Mx[i]+ (1-ld)*muM;}

else{Zp[i]=ld*Mx[i]+(1-ld)*Zp[i-1];}

sigmp[i]=(Zp[i]/(1-(1-ld)^i))

sigmt[i]=abs(sigmp[i])

if(sigmt[i] > 0 && sigmt[i] <= 1)

{

sci = 1/(a*(1+(sigmt[i])^(-2)))

}

else if(sigmt[i] > 1 && sigmt[i] <= 2.7)

{

sci = 1/(a*(1+(sigmt[i])^(-1)))

}

else

{

sci=1.00

}

if(i==1)

{Wp[i]=sci*Mx[i]+ (1-sci)*muW;}

else{Wp[i]=sci*Mx[i]+(1-sci)*Wp[i-1];}

if(abs(Wp[i])>h)

{rl[j]=i;break;}

else{rl[j]=0;}

}

}

mean(rl)

sd(rl)
